# Coupling antecedent rainfall for improving the performance of rainfall thresholds for suspended sediment simulation of semiarid catchments

**DOI:** 10.1038/s41598-022-08342-6

**Published:** 2022-03-21

**Authors:** Zhaorui Yin, Guanghua Qin, Li Guo, Xuan Tang, Jinxing Wang, Hongxia Li

**Affiliations:** 1 State Key Laboratory of Hydraulics and Mountain River Engineering, College of Water Resource and Hydropower, Sichuan University, Chengdu, 610065 China; 2 Information Center (Hydrology Monitor and Forecast Center), Ministry of Water Resources of the People’s Republic of China, Beijing, 100053 China

**Keywords:** Environmental sciences, Hydrology

## Abstract

Suspended sediment transport is one of the essential processes in the geochemical cycle. This study investigated the role of rainfall thresholds in suspended sediment modeling in semiarid catchments. The results showed that rainfall-sediment in the study catchment (HMTC) could be grouped into two patterns on the basis of rainfall threshold 10 mm. The sediment modeling based on LSTM model with the rainfall threshold (C-LSTM scheme) and without threshold (LSTM scheme) were evaluated and compared. The results showed that the C-LSTM scheme had much better performances than LSTM scheme, especially for the low sediment conditions. It was observed that in the study catchment, the mean NSE was marginally improved from 0.925 to 0.934 for calibration and 0.911 to 0.924 for validation for medium and high sediment (Pattern 1); while for low sediment (Pattern 2), the mean NSE was significantly improved from -0.375 to 0.738 for calibration and 0.171 to 0.797 for validation. Results of this study indicated rainfall thresholds were very effective in improving suspended sediment simulation. It was suggested that the incorporation of more information such as rainfall intensity, land use, and land cover may lead to further improvement of sediment prediction in the future.

## Introduction

Suspended sediment transport is a very important part of both the catchment and global geochemical cycle^1^, which has a great influence on many respects such as pollution and degradation^2–4^. So suspended sediment modeling is crucial for catchment management and operation of water resources projects^5–7^. However, the transportation of sediment is a complex process, which includes fluid-sediment interaction and the characteristics of both flow and sediment. This makes the sediment modeling quite difficult by using physically-based models, which are high computational costs and have a high demand for input data^5,8,9^.

The AI (Artificial Intelligence) and computational approaches such as artificial neural networks (ANNs) have the effective ability with regard to the high non-linear nature and complexity of the employed data^10,11^. It has been developed and widely used for hydrologic modeling recently^12–16^. Specifically, in the field of sediment prediction and forecasting, there are many AI techniques and methods that have been used, such as artificial neural network (ANN), support vector machine (SVM), fuzzy logic (FL), and numerous search optimization and statistical learning method^12–16^. For example, Kisi et al.^17^ modeled the suspended sediment using genetic programming. Cobaner et al.^18^ estimated suspended sediment concentration by adaptive neuro-fuzzy and neural network approaches. Singh and Panda^19^ simulated daily suspended sediment load using artificial neural networks and cross validation method for a small agricultural watershed.

Long Short-Term Memory (LSTM) networks are a special type of recurrent neural networks with an internal memory, which has the ability to learn and store long-term dependencies of the input–output relationship^20,21^. So it has great capability in capturing highly complex data distributions for predictions than traditional neural networks without explicit cell memory^10,20,22,23^. Such a superiority and efficiency of the LSTM model has been reported in the fields of flood forecasting^24^, runoff modeling^20^, groundwater level simulation^25^, and sediment modeling^26–28^. Kratzert et al.^20^ simulated rainfall-runoff and the results revealed that LSTM could recognize the long-term relation of inputs and targets. Nourani and Behfar ^28^ proposed two new seasonal-based LSTM models for runoff-sediment modeling, and the results showed that the models had good performances. Huang et al.^22^ adopted LSTM and other neural networks for real-time forecasting of suspended sediment concentrations reservoirs, and the results showed that LSTM was superior to those other machine learning models. Kaveh et al.^29^ evaluated the efficiency of LSTM model in estimating suspended sediment concentration in a river in the United States, and it indicated that LSTM model led to more accurate results.

Although LSTM network can extract the complex relationship patterns of data, yet it still could not effectively capture data with thresholds. Thresholds and other non-linear behaviors are quite common inhydrologic and geomorphic systems^30^, and they can occur at different levels of complexity and may limit the predictability of hydrological processes^31–33^. Rainfall threshold is one of the most key controlling factors for runoff, sediment and landslide^33–39^. For example, Guzzetti et al.^40^ studied the rainfall thresholds for the initiation of landslides worldwide. Western and Grayson ^41^ found that surface runoff was a threshold process controlled by catchment wetness conditions. Castillo et al.^34^ explored the role of antecedent soil water content in the runoff response of semiarid catchments, and results showed that the antecedent soil water content was important for controlling runoff during medium and low-intensity storms.

The role of rainfall threshold and soil moisture was found especially obvious in semiarid and arid environments^42,43^.Soil erosion is accelerated on land where erosive rain falls on landscapes and an obvious sediment process will occur when rainfall over some threshold^4,44,45^. Different runoff and sediment will produce under different rainfall conditions^46^. Meng et al.^44^ explored the impact of rainfall patterns on the soil loss of the hillslope, and results showed that the soil erosion was quite different under moderate, heavy and storm rainfall patterns.

The Loess Plateau, which is located in the arid/semiarid regions of North China, is highly fragmented by gullies and has suffered severe soil erosion^47–49^.The catchment in this study (Heimutouchuan, HMTC) is a small semiarid catchment and flows down the Loess Plateau. It is noticed that the suspended sediment transport rate would increase when a certain rainfall amount is exceeded, which indicated the inherent rainfall-sediment mechanism and relationship changes when reaching or exceeding some rainfall threshold. So it is necessary to explore the rainfall-sediment patterns and the role of rainfall threshold in sediment prediction.

The main objective of this study is to improve the suspended sediment prediction of semiarid catchments through (1) exploring the rainfall thresholds based on rainfall both on the given day and antecedent days, (2) integrating rainfall thresholds in the LSTM model for improving sediment modeling. This study is expected to provide a better understanding and modeling of the sediment processes of semiarid catchments.

## Methodology

### Study area and data

**Figure 1:**
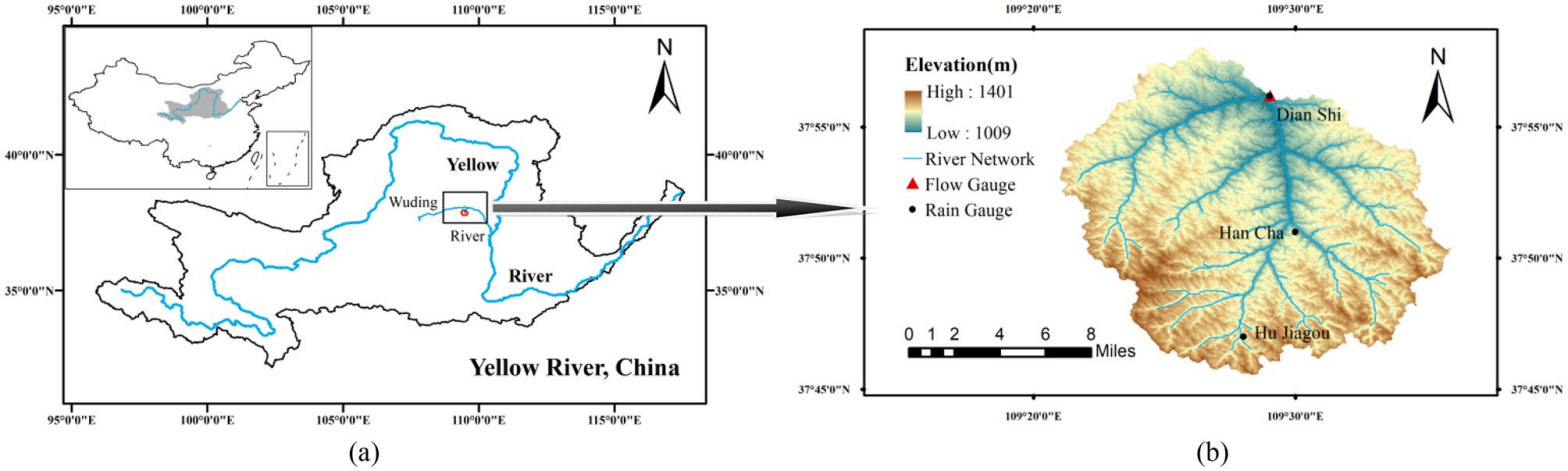
Location of the study area and stations (map generated using ArcMap 10.4(10.4.1)^50^ using ASTER GDEM digital elevation model 30 m resolution^51^).

HMTC catchment is a tributary river of the Yellow River and flows down the Loess Plateau. Figure 1 shows location of the catchment. HMTC lies between 109°15′–109°37′ E longitude and 37°44′–38°00′ N latitude. The location of rain and flow gauges is also showed in Fig. 1. There are three rain gauges and one flow (sediment) gauge in HMTC catchment. Data used in this study are daily runoff (*Q*_*t*_), daily areal rainfall (*P*_*t*_, weighted by Thiessen polygon method for the three rainfall stations) and daily suspended sediment transport rate (*S*_*t*_) in 1980–2010 (July to October).

**Figure 2:**
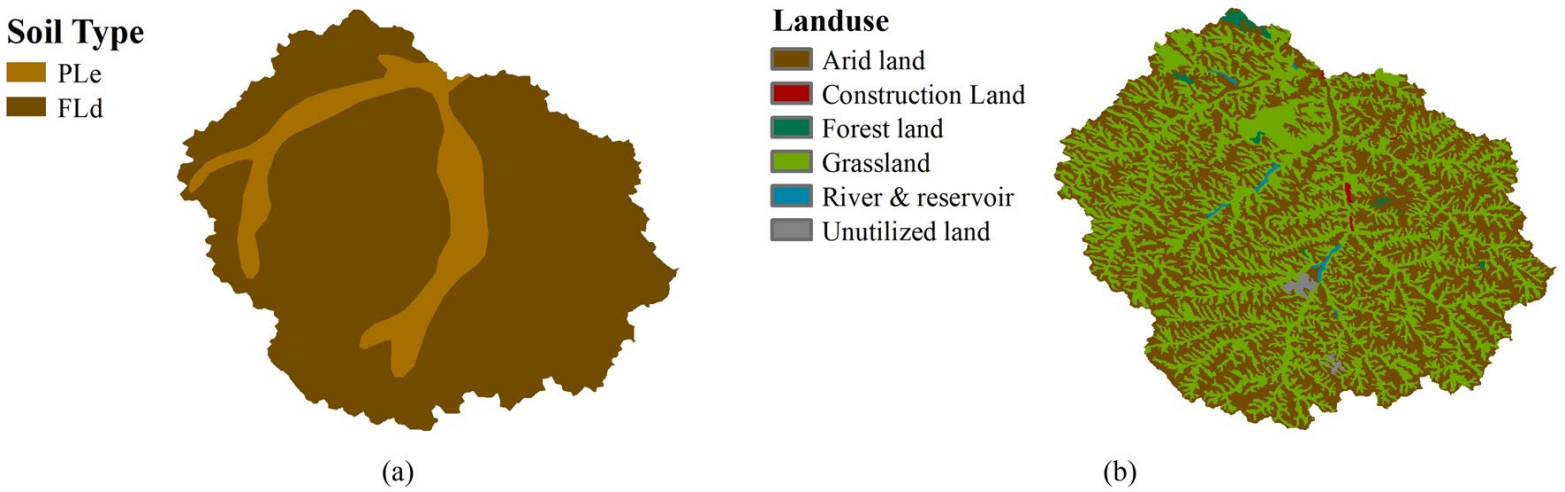
Soil type (**a**) and land use (**b**) of the study area.

The catchment area of HMTC is 327km^2^ and the characteristics of the catchment for the present study are given in Table1. The average annual rainfall is 260 mm and the average annual temperature is 8.6 °C. The daily average runoff is between 0.1 and 67.1 m^3^/s, and the daily sediment transport rate changes widely from 0.001 to 30,100 kg/s, which mainly occurs in summer (July to August). The soil type and land use are shown in Fig. 2. The soil type of HMTC is mainly PLe and FLd (FAO90, HWSD) (Fig. 2). The area contribution of the HMTC catchment is 52.76% under arid land, 45.72% under grass cover, 0.54% under forest land, 0.14% under other construction land, and 0.45% for under unutilized land with a remaining 0.38% underwater bodies for the year (Table 2).

**Table 1:** Characteristics of the study catchment.

Area (km^2^)	Elevation (m)			Average slope (°)	Average annual rainfall (mm)	Average annual temperature (°C)	Daily runoff (m^3^/s)		Daily sediment transport rate (kg/s)	
	Max	Min	Mean				Max	Min	Max	Min
327	1401	1009	1201	11.95	260	8.6	67.4	0.1	30,100	0.001

**Table 2:** Soil and land use types in the study area.

Categories	Soil type		Land use					
	Ple	Fld	Arid land	Grass land	Forest land	Construction land	Rivers and Reservoirs	Unutilized land
Percentage	13.52%	86.48%	52.76%	45.72%	0.54%	0.14%	0.38%	0.45%

### LSTM model

**Figure 3:**
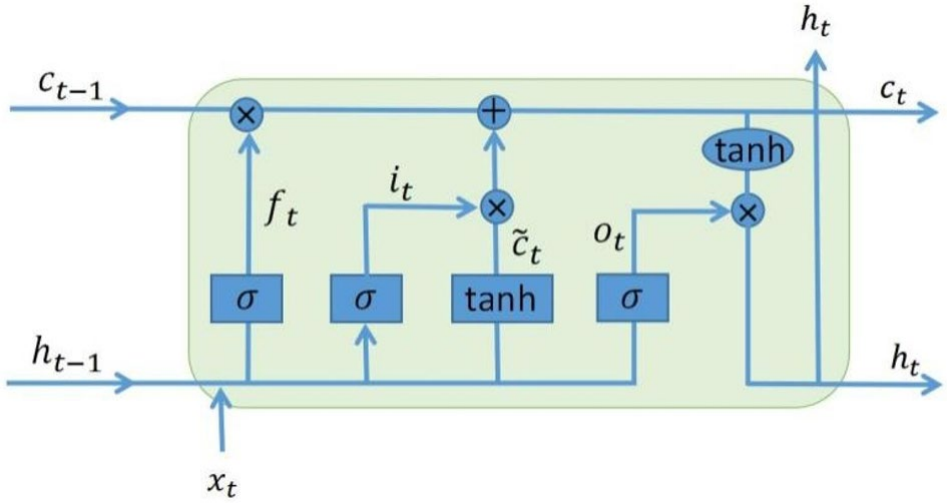
Structure of LSTM neural network model ($f_{t}$, $i_{t}$, and $o_{t}$ are forget gate, input gate and output gate respectively; $\sigma$ and $\tanh$ are activation functions; $\otimes$ is matrix and element product, $\oplus$ is addition).

The LSTM model is one of the deep learning techniques which shows the great ability for dealing with time series problems by considering information selections and long-term dependencies^21^. LSTM can capture highly complex data distributions through memory units (Fig. 3), composed of a forget gate, an input gate and an output gate. The addition of the memory unit in the hidden layer enables the LSTM to learn the state characteristics of the long-period sequence data, making the memory information in the time series controllable, thereby solving the problem of the disappearance or explosion of the traditional RNN (Recurrent Neural Network) gradient^10,20^.

LSTM introduces a new internal state variable $c_{t}$ dedicated to linear cyclical information transmission, and at the same time non-linearly outputs information to the external state of the hidden layer $h_{t}$, $c_{t}$ and $h_{t}$ the calculation formula is as follows:

1 $$c_{t} = f_{t} \otimes c_{t - 1} + i_{t} \otimes \tilde{c}_{t}$$

2 $$h_{t} = o_{t} \otimes \tanh (c_{t} )$$

$\tilde{c}_{t}$ is the candidate state variable obtained by the nonlinear function:

3 $$\tilde{c}_{t} = \tanh (W_{c} x_{t} + U_{c} h_{t - 1} + b_{c} )$$

LSTM introduces a gating mechanism to control the path of information transmission. The calculation formula for the three gates $i_{t}$, $f_{t}$, $o_{t}$ and the memory unit are:

4 $$i_{t} = \sigma (W_{i} x_{t} + U_{i} h_{t - 1} + b_{i} )$$

5 $$f_{t} = \sigma (W_{f} x_{t} + U_{f} h_{t - 1} + b_{f} )$$

6 $$o_{t} = \sigma (W_{o} x_{t} + U_{o} h_{t - 1} + b_{o} )$$

LSTM network introduces the gating mechanism to control the path of information transmission, the cell state vector ensures a continuously updated long-term memory^52,53^. In such a method, the forget and input bits respectively decide (i) whether to reset the cell states from the previous time-stamp and forget the past and (ii) whether to increment the cell states from the previous time-stamp to incorporate new information into long-term memory^52,53^. Therefore, in the memory unit, not only a piece of certain key information can be captured at sometime through the forget gate, and the key information can be saved for a certain time interval, but also the historical information can be directly emptied through the forget gate. In this way, the LSTM neural network can selectively retain or forget previous information.

In this study, the rainfall thresholds are integrated in the LSTM model for improving sediment modeling.The sediments modeling is developed based on LSTM model without threshold (LSTM scheme) and with the rainfall threshold (C-LSTM scheme). The training of C-LSTM after data classification means a part of forgetting and selection in advance according to the mechanism. For example, when the input and output data corresponding to high sediment appears in the model operation, the previous low sediment content will be forgotten, and the high sediment content retains more information. In this way, the C-LSTM method would get the useful information and not miss the essentials.

### Antecedent rainfall model

Rainfall threshold is affected by rainfall on the given day and antecedent days. Antecedent rainfall is usually estimated by an empirical approach through an index (the Antecedent Precipitation Index, API) based on the cumulated rainfall with a short period preceding the event^50^. Several antecedent rainfall models have been proposed^52,53^, which are as follows:

7 $$P_{a,t} = KP_{t - 1} + K^{2} P_{t - 2} + \cdots + K^{n} P_{t - n}$$

8 $$P_{a,t} = P_{t - 1} + 2^{d} P_{t - 2} + 3^{d} P_{t - 3} + \cdots + n^{d} P_{t - n}$$

where ${P}_{a,t}$ is antecedent rainfall (mm) for day t; *K* is a constant coefficient, usually about 0.8–0.9; ${P}_{t-n}$ is the rainfall (mm) on the nth day before 0, and *n* is usually 5-15d; *d* is the recession curve coefficient, which can be derived from the hydrograph (*d* < 0)^51^.

Equation (8) considers the fast drainage of soils and results in a shift of the distribution of the daily rainfall magnitudes to lower antecedent daily rainfall values^54^. HMTC catchment is located in the semiarid areas and does not have large long-term water storage capacity, so the Eq. (8) with lower antecedent daily rainfall is more appropriate for estimation of soil moisture conditions in the study catchment.

The recession curve coefficient *d* has a strong influence on the magnitude of antecedent rainfall. The larger the absolute value of the exponent, the faster water drains from the soil, thus lowering the time interval of effective antecedent rainfall influence to the critical water content required to sediment.

### Evaluation of model performances

Nash–Sutcliffe efficiency (NSE), the coefficient of determination (R^2^), root mean square error (RMSE), and relative bias (BIAS) are used to evaluate the accuracy of sediment simulation results, which are defined as follows:

9 $$NSE = 1 - \frac{{\sum {(S_{obs} (i) - S_{sim} (i))^{2} } }}{{\sum {(S_{obs} (i) - S_{obs,mean} )^{2} } }}$$

10 $$R^{2} = \frac{{\left( {\sum\nolimits_{i = 1}^{n} {(S_{obs} (i) - S_{obs,mean} )(S_{sim} (i) - S_{sim,mean} )} } \right)^{2} }}{{n\sum\nolimits_{i = 1}^{n} {(S_{obs} (i) - S_{obs,mean} )^{2} \cdot } \sum\nolimits_{i = 1}^{n} {(S_{sim} (i) - S_{sim,mean} )^{2} } }}$$

11 $$RMSE = \sqrt {\frac{{\sum\nolimits_{i = 1}^{n} {(S_{obs} (i) - S_{sim} (i))^{2} } }}{{S_{mean} }}}$$

12 $$BIAS = \frac{{\sum\nolimits_{i = 1}^{n} {\left| {S_{sim} (i) - S_{obs} (i)} \right|} }}{n}$$

where $S_{obs}$ and $S_{sim}$ are observed and modeled daily sediment respectively, $S_{obs,mean}$, $S_{sim,mean}$ are the arithmetic mean of the observed and modeled daily sediment, $i$ is the *i*th sample, and *n* is the number of samples.

## Results and discussion

### Rainfall threshold and rainfall pattern

**Figure 4:**
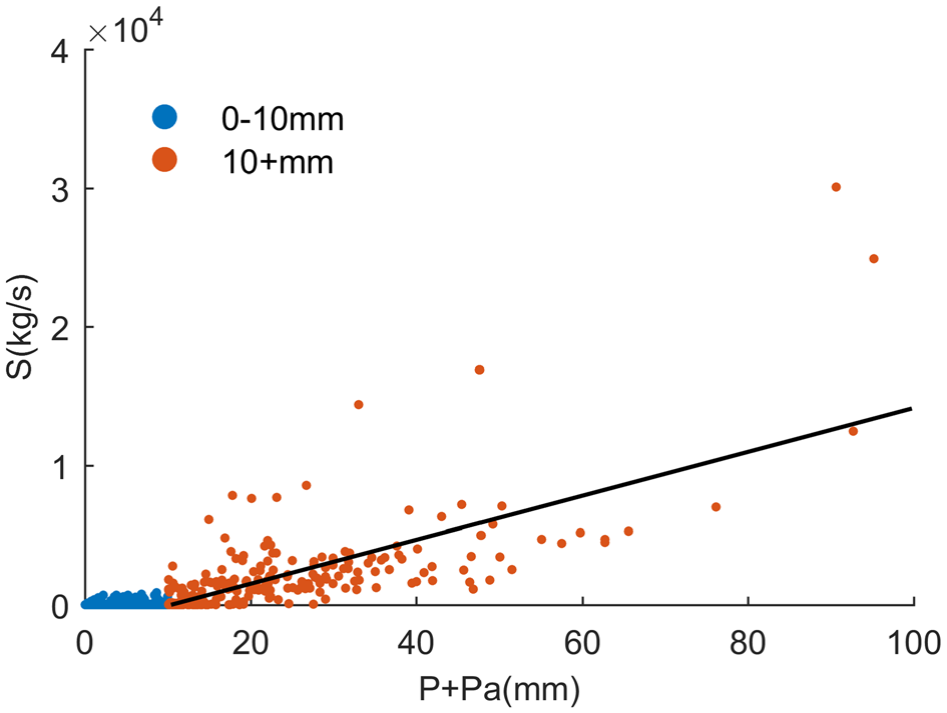
Relationship of rainfall (*P* + *P*_*a*_) to sediment (*S*).

To derive rainfall thresholds, the rainfall-sediment relationship under different rainfall conditions was investigated to identify patterns in behaviors. Both rainfall on the given day (*P*) and antecedent days (*P*_*a*_) were considered to link to the sediment (Fig. 4). Antecedent rainfall of HMTC was estimated by Eq. (8). After calculation, the recession curve coefficients, i.e., *d* = − 1.65, and *n* = 5 days, were used in this study for HMTC catchment.

Figure 4 displays the relationship between sediment rate *S*, and rainfall *P* + *P*_*a*_. The red dots were related to rainfall (*P* + *P*_*a*_) > 10 mm, and the blue dots relating to rainfall (*P* + *P*_*a*_) < 10 mm. It was noticed that rainfall-sediment relationships undergone changes with rainfall over 10 mm. This indicated sediment process would change when rainfall exceeds 10 mm threshold. So the rainfall-sediment relationships can be mainly grouped into two patterns by rainfall threshold 10 mm. Pattern 1 was associated with medium and high daily rainfall (*P* + *P*_*a*_ > 10 mm) mainly leading to medium and high suspended sediment, while Pattern 2 was low rainfall (*P* + *P*_*a*_ < 10 mm) leading to low sediment.

### Comparison of the two schemes for suspended sediment prediction

The sediment modeling in this study is developed based on LSTM model without threshold (LSTM scheme) and with the rainfall threshold (C-LSTM scheme). The same data set were used in both LSTM and C-LSTM schemes for acomparative study. C-LSTM scheme modeled high sediment and low sediment separately, and LSTM scheme simulated all sediments. *Q*_*t*_, *P*_*t*_ and *P*_*a,t*_ were used as input data for modeling in this study. Data of 20 years (1980–1999) were used for model calibration, and data of 11 years (2000–2010) were used for validation.

The performances of the two schemes were examined using the calibration and validation data set. The obtained results of the C-LSTM were compared to LSTM scheme for evaluating the predictive capability. The NSE values of the two schemes are presented in Table 3. It was observed that the C-LSTM scheme showed better performances in predicting the daily sediment in the study catchment. In Pattern 1 for medium and high sediment simulation, the NSE of LSTM was 0.925 and 0.911 for calibration and validation, while the NSE using C-LSTM was 0.934 and 0.924, respectively (Table 3). The results showed the simulation was marginally improved.

**Table 3:** NSE evaluation of simulation results of two schemes.

Catchment	Pattern 1 (medium and high sediment)				Pattern 2 (low sediment)			
	LSTM		C-LSTM		LSTM		C-LSTM	
	Calibration	Validation	Calibration	Validation	Calibration	Validation	Calibration	Validation
HMTC	0.925	0.911	0.934	0.924	− 0.357	0.171	0.738	0.797

Moreover, in Pattern 2 with rainfall *P* + *P*_*a*_ < 10 mm for low sediment simulation, the improvement was more significant. It was observed that the LSTM scheme was unable to capture the low suspended sediment rate as it was very clear a negative NSE value was predicted during low sediment data. The NSE of LSTM was -0.375 and 0.171 for calibration and validation, and was improved to 0.738 and 0.797 by C-LSTM. Results suggested that the C-LSTM scheme was much better than LSTM scheme based on NSE as performance evaluation criteria.

**Figure 5:**
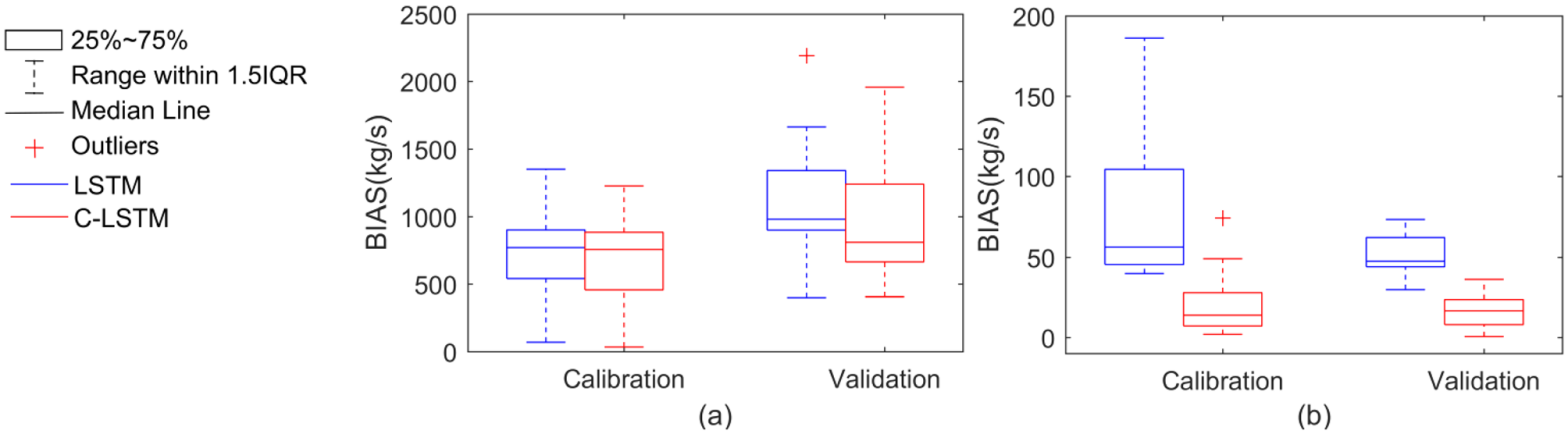
Box plot of BIAS indicator for Patten1 (**a**) and Patten2 (**b**).

Figure 5 and Table 4 further compared simulation results for the calibration and validation periods in terms of BIAS. The median BIAS in Pattern 1 for the two schemes (LSTM and C-LSTM) was 771.7 kg/s and 757.9 kg/s for calibration, as well as, 968.7 kg/s and 811.1 kg/s for validation, respectively; The median BIAS in Pattern 2 for the two schemes was 56.3 kg/s and 12.4 kg/s for calibration, while 47.4 kg/s and 16.7 kg/s for validation, respectively. The BIAS results also suggested that the C-LSTM scheme was better than the LSTM scheme.

**Table 4:** BIAS evaluation for the study catchment (Q_1_, the lower quartile; Q_2_, the median; and Q_3_, the higher quartile).

Evaluation indicators	Pattern 1				Pattern 2			
	LSTM		C-LSTM		LSTM		C-LSTM	
	Calibration	Validation	Calibration	Validation	Calibration	Validation	Calibration	Validation
Range	[69.8–1,349.3]	[400.5–2,191.1]	[34.2–1,225.2]	[407.4–1,957.4]	[39.8–186.23]	[29.7–73.3]	[2.0–74.3]	[0.6–36.1]
Mean	782.7	1,009.9	724.1	983.6	79.5	50.5	17.6	17.0
Q1	547.2	901.4	473.5	707.4	45.7	44.1	7.2	8.2
Q2	771.7	968.7	757.9	811.1	56.3	47.4	12.4	16.7
Q3	901.4	1,151.7	865.7	1,046.8	103.6	60.2	26.4	22.9

**Figure 6:**
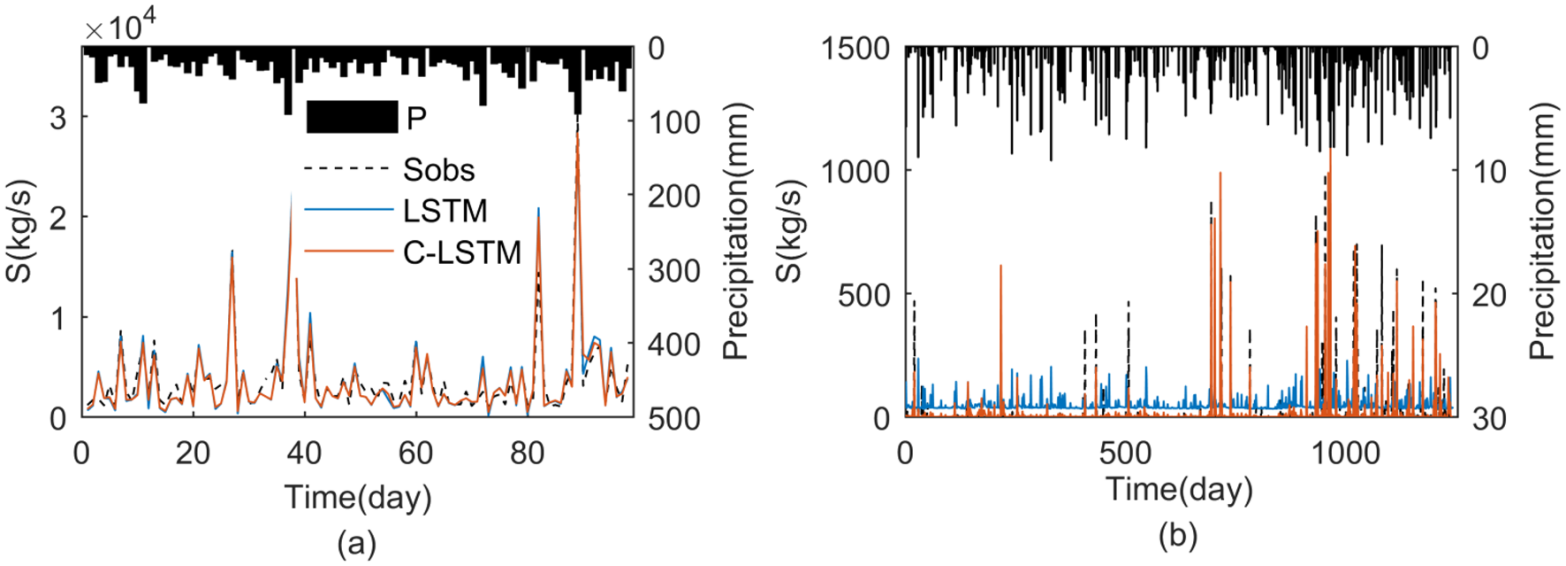
Comparison between observed and estimated sediment using the two schemes for Patten1 (**a**) and Patten2 (**b**).

Further comparisons of the two schemes are shown in the form of a hydrograph in Fig. 6. In pattern 1 for medium and high sediment (Fig. 6a), the hydrographs indicated that the modeled suspended sediment rate by both models followed the variation in the observed data. In pattern 2, the C-LSTM scheme results showed much better performance than that of LSTM scheme. It was seen from the hydrograph that observed and model sediment yielded by LSTM scheme was not followed closely, and the hydrographs indicated that LSTM scheme overestimated the sediment in pattern 2 during the low rainfall days (Fig. 6b).

**Figure 7:**
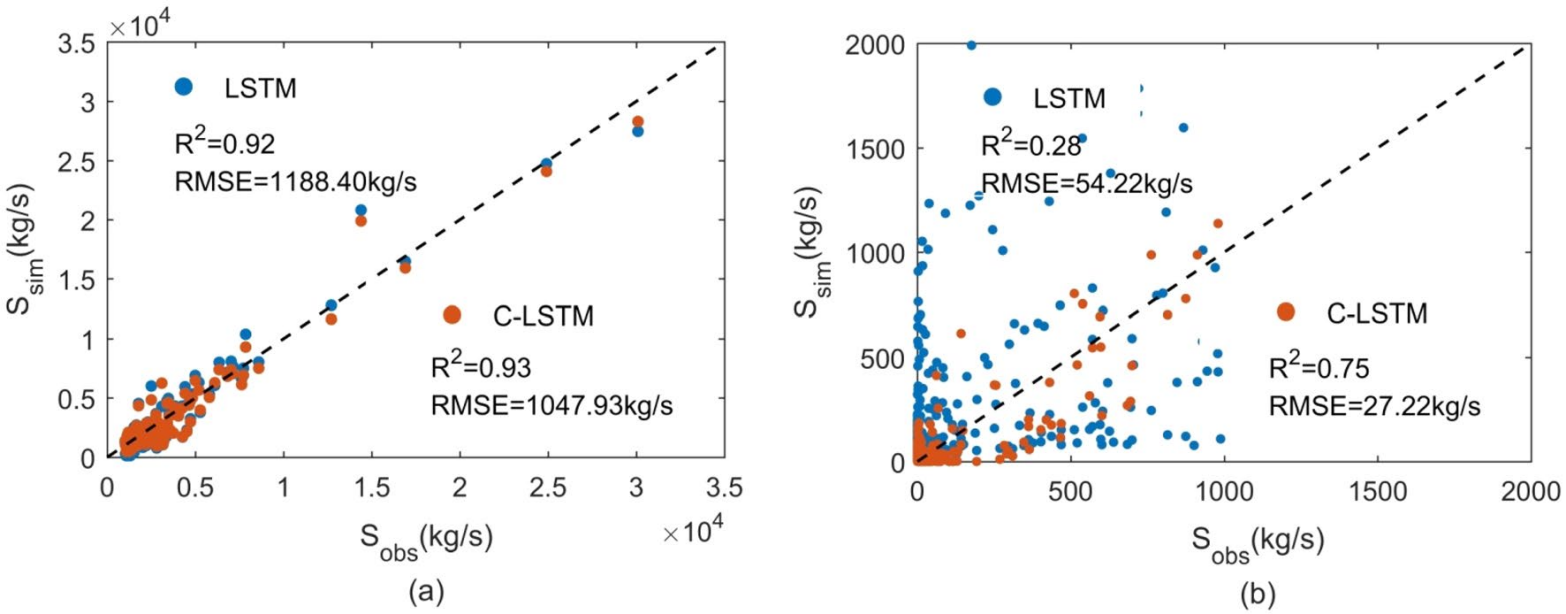
Scatter plot of observed and simulated of sediment for Patten1 (**a**) and Patten2 (**b**).

Similarly, in the scatter plot, it was observed that results in pattern 1 were closer to the 1:1 line, and the data points were scattered around the 1:1 line (Fig. 7a). The RMSE between the observed and modeled sediments obtained from the C-LSTM scheme (1047.93 kg/s) was less than that from LSTM scheme (1188.40 kg/s), and the R^2^ was raised from 0.92 to 0.93. Results exhibited that the C-LSTM scheme slightly outperformed LSTM scheme for medium and high sediment simulation. In Pattern 2 (Fig. 7b), the RMSE from the C-LSTM scheme (27.22 kg/s) was less than that from LSTM scheme (54.22 kg/s). The R^2^ was raised from 0.28 to 0.75, which also suggested that C-LSTM scheme was much better than the LSTM scheme for low sediment simulation.

### Limitations and uncertainties

Results in this study showed that rainfall threshold was a potentially useful factor for sediment modeling in semiarid areas such as the Loess Plateau of China. However, there were still some limitations and uncertainties if only rainfall amount was considered in suspended sediment modeling. For example, there were still some events not following the rainfall threshold (Fig. 4). This was mainly because there were some other factors which may influence the sediment process such as rainfall intensity (PI)^48,55^.

The role of rainfall intensity was analyzed and displayed in Table 5. It showed that some high-intensity rainstorms produce high sediment. For example, the rainfall amount in HMTC on August 8, 1982 (P = 10.01 mm, Pa = 1.72 mm) and September 27, 1984 (P = 10.20 mm, Pa = 1.69 mm) was almost the same, but the former had a greater PI (10.5 mm/h) which led to the higher sediment (1190 kg/s), while PI of the latter was 3.9 mm/h, leading to a lower sediment flux (81.7 kg/s) (Table 5a). This demonstrated high-intensity rainfall would increase soil erosion and sediment response. Another comparison was made between July 7, 2000 and July 19, 1999 (Table 5b). The former had a lower rainfall amount (27.65 mm) but higher sediment (3130 kg/s) because of the higher PI (30.6 mm/h), while the later led to a lower sediment flux (2310 kg/s) because of the lower PI (11.5 mm/h). These indicated that the rainfall intensity had great effects on sediment, and including rainfall intensity as a key factor would be helpful for the reduction of sediment predictive uncertainty. Also, there were still some other factors such as land use and land cover, which may influence the sediment and need to be further considered in the future^55^.

**Table 5:** Relationship between sediment, rainfall amount and rainfall intensity (hourly).

No.	Date	S (kg/s)	P (mm)	Pa (mm)	PI (mm/h)
(a)	1982/8/8	1190	10.01	1.72	10.5
	1984/9/27	81.7	10.2	1.69	3.9
(b)	2000/7/7	3130	26.09	1.56	30.6
	1999/7/19	2310	39.37	1.55	11.5

P refers to the accumulated rainfall in a day; PI is the maximum rainfall intensity (mm/h) of 1 h.

## Conclusions

This study investigated the effectiveness of integrating rainfall thresholds in the sediment modeling in a small semiarid catchment in China. The results showed that coupling antecedent rainfall could lead to better rainfall thresholds. Evaluation of the accuracies of results produced by the C-LSTM and LSTM scheme showed that C-LSTM had much better performances for predicting sediment, especially for low rainfall conditions. This demonstrated the C-LSTM scheme had a better suspended sediment simulation capability compared to LSTM scheme, which indicated the advances of integrating rainfall thresholds for suspended sediment modeling. The results highlighted the importance of integrating rainfall thresholds in sediment prediction in semiarid areas such as the Loess Plateau of China. However, the rainfall intensity, land use, and land cover, which were also very important for the sediment processes, have not been incorporated in the present study and will be included in future research to further improve the estimation capability of suspended sediment.
